# The Influence of a Predegenerated Autological Nerve Graft on the Results of Peripheral Nerve Repair in the Upper Extremities After Injuries

**DOI:** 10.3390/bioengineering12090945

**Published:** 2025-08-31

**Authors:** Krzysztof Suszyński, Bartłomiej Błaszczyk, Dariusz Górka, Stanisław Kwiek

**Affiliations:** 1 Department of Sports Medicine and Exercise Physiology, Medical University of Silesia, 40-055 Katowice, Poland; dgorka@sum.edu.pl; 2 Department of Neurosurgery, Medical University of Silesia, 40-055 Katowice, Poland; 3 Department of Spine Surgery, KCM Clinic SA, 58-500 Jelenia Góra, Poland

**Keywords:** nerve reconstruction, autologous graft, nerve regeneration

## Abstract

The improvement in peripheral nerve repair is still challenging, with the process being difficult and frequently unsatisfying. Injuries, even minor ones, can lead to limitations, including the loss of important life functions such as fingers, hands, or all limbs. Our previous research on animals revealed that the distal part of autologous predegenerated nerve grafts, which were injured and left in place for 7 days, was more capable of supporting reconstructed nerve regeneration. Little is known about the efficacy of using predegenerated autologous grafts in humans. Encouraged by promising results in animal models, we decided to investigate this process in humans. A total of 31 patients were evaluated in the study; 19 predegenerated (injured and left in situ for 7 days) autologous sural nerve implants and 12 fresh sural nerve implants were used, and the period of 2 years after operation was chosen as the time of final clinical assessment. Clinical assessment and motor and sensory nerve conduction velocity were assessed. All data were statistically analyzed using stepwise regression testing and a one-way analysis of variance followed by Tukey’s test for continuous values and the Mann–Whitney U test for ordinal values. Differences were considered statistically significant for *p* ≤ 0.05. It turns out that autologous, predegenerated sural nerve grafts used for the reconstruction of traumatic peripheral nerves results in better quantitative and qualitative clinical functional outcomes and more adequate nerve conduction parameters.

## 1. Introduction

The improvement in peripheral nerve repairs is still challenging, with the process being difficult and frequently unsatisfying. A lot of injuries, sometimes even as an effect of a trivial injury, can lead to the loss of important functions of the fingers, hand, or limbs. The main cause of loss is the necessity of the mobilization of nerve stumps [1,2,3,4]. Reconstruction of the skin using nerve implants does not lead to tension in the junction line and enables correct provision with blood in the reconstructed nerve. This forms the basis for sufficient regeneration [5]. The autologous grafts enable early rehabilitation and minimize the risk of expansion-related secondary injury. Many reports on the good results of treatment with autological grafting have been published [6,7,8,9]. Understanding the pathomechanisms behind peripheral nerve damage and learning the course of regeneration seem to be crucial for selecting the appropriate methods of treatment. The gold standard of treatment for peripheral nerve gaps between 5 mm and 3 cm in size is the nerve conduit or autologous nerve graft, interchangeably; however, this last treatment is often associated with a variety of clinical complications. Predegenerated grafts have been suggested to be responsible for faster regeneration in animal models by reducing the initial delay period for nerve regrowth [10,11]. The concept that multiple heterogeneous neurotrophic factors play a vital role in regeneration processes seems logical. Faster growth of mobile Schwann cells is also very important [12,13,14]. Our previous studies on animals [15,16,17] have shown that 7 days after predegeneration of the donor nerve, its distal part has an optimal ability to support the reconstructed nerve regeneration. Electrophoresis of the postmicrosomal fractions obtained from human peripheral nerves revealed quantitative and qualitative changes in the protein’s composition. In all predegenerated nerves, the total protein concentration was significantly higher (*p* < 0.05) than in the fragments obtained from the proximal stump of the injured nerve. The analyzed protein mixtures were resolved into 41 bands whose apparent molecular weight ranged from 14.9 to 354.4 kDa in one-way SDS-PAGE electrophoresis [15]. In this range of molecular weights, neurotropic and neurotrophic factors such as NGF, BDNF, CNTF, IGF-I, IGF-II, and proteins derived from myelin degradation, such as S-100, PLP, and Wolfgram proteins, were found [12,13,14]. This condition is attributed to the increased proliferation of Schwann cells due to Wallerian degeneration after transection of a peripheral nerve and related augmentation in the secretion of NGF, which combine to support axonal regeneration in the PNS. Little is known about the efficacy of using predegenerated grafts in humans [18]. Encouraged by promising results in animal models, we decided to investigate this process in humans.

## 2. Materials and Methods

Patients hospitalized in the Neurosurgery Department of the Medical University of Silesia in Katowice were investigated. In total, 31 persons were examined. Ethical approval was obtained from the Regional Ethics Review Committee, Katowice, Poland (register number 1-208/05). The average age was 36.2 years (min.15–max.60). The average length of autologous grafts was 3.7 cm. The average period from injury to final reconstruction was 18.9 months. The right hand was the predominant hand in 20 (64.5%) persons and left was predominant in 11 (35.5). The average period of observation from the operation to the last examination was 28.5 months. Damage concerned the ulnar nerve in 58.1% (n = 18), the median nerve in 38.7% (n = 12), and the radial nerve in 3.2% cases (n = 1). In the group of patients with a presence of collateral reinnervation, the period between injury and final reconstruction was 20.8 months and was comparable with the group without reinnervation (23.6 months).

Predegenerated nerve graft was examined as a method of nerve injury surgery in the Neurosurgery Department, Medical University of Silesia. The sural nerve (the graft) was cut 7 days before reconstruction. Its distal section was marked and left in situ until the final reconstruction procedure. Microsurgical reconstruction of the injured antebrachial nerve was performed after 7 days. Distal and proximal stumps of the reconstructed nerve were localized and refreshed; epineurum was cut to show individual fascicles. The predegenerated sural nerve was collected, and two or three nerve cables were implanted and provided with perineural suture (10.0 monofilat), with four to six stiches without tension (see Supplementary Materials).

Exclusion criteria were as follows: age under 17 years old, neurological comorbidities, past surgery in injured upper extremities, and mental/psychological disorders.

All patients were divided into two groups on the basis of the surgical reconstruction of the damaged nerve method, including (a) the traditional method using the fresh sural nerve implant—(T), (n = 12 persons) and (b) the new method using the predegenerated sural nerve implant—(P), (n = 19 persons). The collateral reinnervation occurrence included (a) the group with reinnervation present (R), (n = 8 persons) and (b) the group without reinnervation (NR), (n = 23 persons). The assignment into (R) and (NR) groups was performed on the basis of the electromyography tests.

The volume of the hand up to the distal-most wrist crease was measured. In this method, the limb is submerged into a water tank, and the volume of water displaced is assumed to be equal to the limb volume. The volume of the impaired hand was expressed as a percentage of the healthy hand’s volume (hand volume index—HVI). To evaluate muscle strength, a dynamometric measurement method using a baseline hand dynamometer was employed. Muscle strength of the impaired hand was expressed as a percentage of the healthy hand’s strength (hand muscle strength index—HMSI). To evaluate autonomic changes in the impaired hand, a unique scale was created. The following characteristics were analyzed: sweating disorders, skin color, temperature, and humidity in the area provided by the reconstructed nerve. Grading: 1—extensive vegetative changes; 2—noticeable changes; 3—without changes.

The electrophysiological assessment of the peripheral nerve function after surgical treatment was performed with electroneurography tests. Nerve conduction velocity testing (NCV) was used to evaluate any damage or disease in peripheral nerves. Examinations were performed in the Department of Neurosurgery of the Central Clinical Hospital, the Medical University of Silesia in Katowice with the Viking IV D apparatus by Nicolet. The examination consisted of two main parts, namely the motor NCS and sensory NCS recording. The evaluation of the conducted assessment was based on an analysis of the (MNCS_CV)—Motor Nerve Conduction Study_Conduction Velocity, (SNCS_CV)—Sensory Nerve Conduction Study_Conduction Velocity, (MNCS_OL)—Motor Nerve Conduction Study_Onset Latency, (MNCS_APA)—Motor Nerve Conduction Study_Action Potential Amplitude, and (SNCS_APA)—Sensory Nerve Conduction Study_ Action Potential Amplitude. The results were presented as 1: the expansion of conduction; 2: reference of the relative value to an exemplar value for the given type of nerve; and 3: the assessment of conduction parameters according to a 4-grade scale as follows: (grade 1—the absence (or a trace) of an answer; grade 2—a clear answer with abnormal parameters; grade 3—a clear answer with parameters similar to regular ones; grade 4—a clear answer with regular parameters).

All data were statistically analyzed using stepwise regression testing and one-way analysis of variance followed by Tukey’s test for continuous values and the Mann–Whitney U test for ordinal values. Differences were considered statistically significant for *p* ≤ 0.05.

## 3. Results

**Figure 1:**
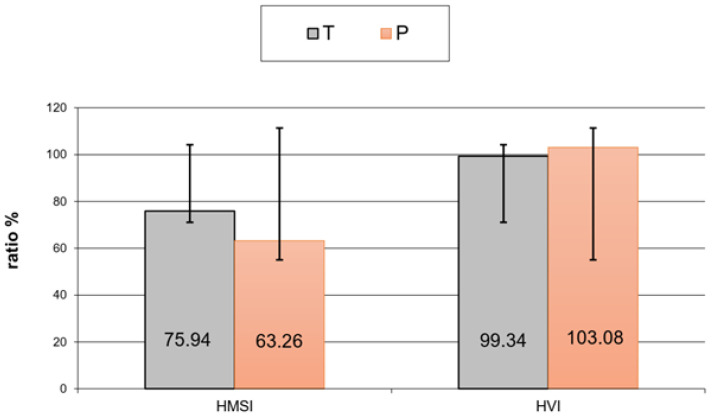
Hand muscle strength index and hand volume index in both groups after regression analysis.

The average period from the injury to the final reconstruction was 12.9 months in the predegeneration (P) group. EMG examination showed that collateral reinnervation from nearby seated, non-injured nerves occurred only in the group with predegenerated graft from undamaged adjacent nerve fibers was present in higher numbers (statistically insignificant) in patients with predegenerated grafts. Predegenerated group HVI results showed better outcomes compared to the T group (*p* = 0.0254) (Figure 1).

**Figure 2:**
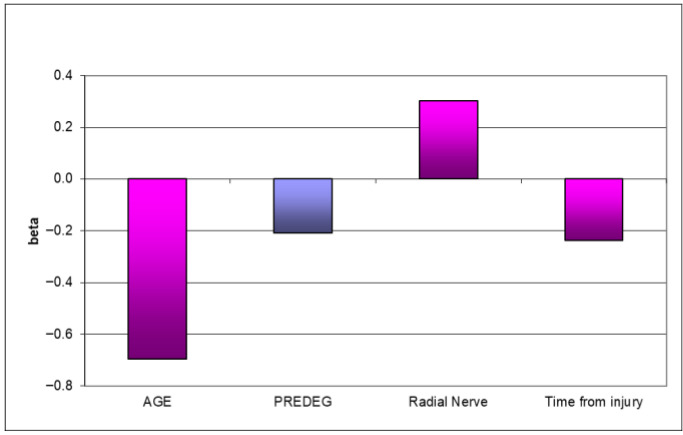
Regression analysis (final step)—functional assessment of injured side. (Violet—Predegenerated group).

A moderate number of autonomic disorders were noticed in 50% of P group patients. This increased as patients underwent the new method of treatment, commonly reporting autonomic abnormalities. A statistically significant positive influence of predegenerated graft employment was observed (*p* = 0.02439) in hand volume (HVI) (Figure 1). Considering all examined parameters, only predegeneration could be proven to be the positive factor in sensory function recovery (*p* = 0.0368). Older age negatively influenced all the obtained results (*p* = 0.0073) (Figure 2).

Electrophysiological examinations of nerve conduction show statistically better results of peripheral nerve reconstruction in the predegeneration group. Referring to the relative value of amplitude, onset latency, and conduction velocity of motor conduction evoked potential (PNR) to an exemplar value for the given type of nerve analysis shows congruent results in group P. The amplitude of the action potential values was even better in P group, as shown in Table 1 (results of the electrophysiological assessment) and Table 2 (the mean values of particular parameters for given type of nerve). The multivariate regression analysis showed that damage relating to the predominant hand essentially favorably influences the range of motor and sensory conduction velocity (*p* = 0.04427) and action potential amplitude (*p* = 0.04651). Moreover, the practical application of predegenerated sural nerve grafts translates to unfavorable results in the amplitude of sensory action potential NCV (*p* = 0.0371).

## 4. Discussion

This investigation evaluated the effects of post-injury peripheral nerve reconstruction with the sural nerve graft cut 7 days in advance and left in situ until sampling. Clinical research concerned improvements in the movement, sensory ability, strength, volume, and autonomic function of implanted nerve grafts, involving electrophysiological examination. In this paper, we evaluated the influence of introductory degeneration of the graft on functional recovery in all examined parameters. The analysis presented above shows benefit in movement and sensory function in patients who underwent the predegenerated graft procedure. Older patients only saw minor progress in sensory and movement outcomes. General strength and volume analyses revealed improvements in the hand volume index—HVI—in patients with predegenerated grafts, while in the hand muscle strength index—HMSI—patients with collateral reinnervation showed better outcomes. The P group patients showed excellent strength and volume progress in their non-dominant hand. When the dominant hand was injured, the P group patients obtained worse results. The regression analyses showed a statistically significant positive influence of predegenerated graft usage on hand volume, and hand strength was correlated with the older age of patients and a long period between injury and reconstruction. Vegetative changes were rarely observed in predegeneration group patients. No autonomic disorders were found in 1/3 of all patients in the P group; however, 50% of P group patients were classified as being in the second grade of clinical improvement [19,20,21,22,23,24,25]. In cases when collateral innervation was observed, even better results were obtained. Electroneurographic examination on movement velocity, latency, movement amplitude, sensory velocity, and sensory amplitude was performed. All patients who underwent the predegenerated graft procedure obtained better results in both movement and sensory parameters after two years of observation. The age of patients and dominant hand injury were the essential factors determining functional recovery time. Collateral reinnervation was the main factor affecting the muscular answer amplitude, emphasizing its influence on early strength recovery. Thumble and co. [24] stated minimal muscle strength differences between dominant and non-dominant extremities after fresh autological graft implantation. In this study, similar results were observed in patients with non-dominant hand injury, while in patients with dominant hand trauma, worse strength results were obtained. The conclusion of a too long period between injury and final surgery being the factor of final outcome deterioration correlates with other research [20,25,26]. The results of peripheral nerve reconstruction are more optimistic in younger patients compared with older ones [1,13]; this conclusion is also found in this experiment. A long gap diminishes movement and sensory recovery when cutaneous nerves implants are used [27,28]. Our research is in accordance with Danielsen et al.’s work [27,28], where predegenerated implants decreased initial delay in injured nerve regeneration. This phenomenon improves final functional outcomes, which was also revealed in this study. Further clinical trials with a high level of reliability need to be conducted. Furthermore, more technologically advanced absorbable conduits or nerve grafts should be used to keep up with progress in bioengineering. Such conduits should include hybrid implants, especially full neural scaffolds that imitate the outer layer of the nerve and the endoneurium [11,29,30,31]. A milestone, especially in repairing nerve gaps of a larger size, might be the enrichment of the internal environment of the conduit by adding mixture of growth factors and adhesion proteins [13,30,31]. However, one must remember that the use of growth factors may cause many undesired side effects because to date, knowledge of their physiological concentrations is still unsatisfactory.

The limitation of this study is the small number of cases on which the findings are based. Therefore, a more extensive study involving larger groups is necessary to attain statistically conclusive results.

## 5. Conclusions

Predegenerated autologous nerve grafts improve nerve regeneration by decreasing the initial delay period, which results in better quantitative and qualitative clinical functional outcomes. Autologous predegenerated sural nerve grafts used for the reconstruction of traumatic peripheral nerve injury result in more adequate conduction NCV parameters. Predegeneration intensifies axonal regeneration and accelerates the electrophysiological results of conduction, evoking potential for better functional recovery of injured nerves.

## Figures and Tables

**Table 1:** The electrophysiological improvement scale of conduction parameters in both groups.

	MNCS_CV	MNCS_CV	MNCS_CV	MNCS_CV
**Group**	**Grade 1**	**Grade 2**	**Grade 3**	**Grade 4**
**T (n)**	3	2	0	7
**%**	25.00%	16.67%	0.00%	58.33%
**P (n)**	1	8	3	7
**%**	5.26%	42.11%	15.79%	36.84%
	**MNCS_OL**	**MNCS_OL**	**MNCS_OL**	**MNCS_OL**
**Group**	**Grade 1**	**Grade 2**	**Grade 3**	**Grade 4**
**T (n)**	3	3	1	5
**%**	25.00%	25.00%	8.33%	41.67%
**P (n)**	1	7	3	8
**%**	5.26%	36.84%	15.79%	42.11%
	**MNCS_APA**	**MNCS_APA**	**MNCS_APA**	**MNCS_APA**
**Group**	**Grade 1**	**Grade 2**	**Grade 3**	**Grade 4**
**T (n)**	3	0	7	2
**%**	25.00%	0.00%	58.33%	16.67%
**P (n)**	5	2	7	5
**%**	26.32%	10.53%	36.84%	26.32%
	**SNCS_CV**	**SNCS_CV**	**SNCS_CV**	**SNCS_CV**
**Group**	**Grade 1**	**Grade 2**	**Grade 3**	**Grade 4**
**T (n)**	5	2	0	5
**%**	41.67%	16.67%	0.00%	41.67%
**P (n)**	4	5	1	9
**%**	21.05%	26.32%	5.26%	47.37%
	**SNCS_APA**	**SNCS_APA**	**SNCS_APA**	
**Group**	**Grade 1**		**Grade 3**	**Grade 4**
**T (n)**	4		2	6
**%**	33.33%		16.67%	50.00%
**P (n)**	5		8	6
**%**	26.32%		42.11%	31.58%

**Table 2:** Individual conduction NCV parameters referring to an exemplar value for the given type of nerve (the mean values).

MNCS_CV (Ulnar Nerve)	Group P	Group T
mean	37.59 m/s	49.9 m/s
MNCS_OL (ulnar nerve)	P	T
mean	3.12 ms	3.24 ms
MNCS_APA (ulnar nerve)	P	T
mean	3.45 mV	3.32 mV
SNCS_CV (ulnar nerve)	P	T
mean	37.86 m/s	40.6 m/s
SNCS_APA (ulnar nerve)	P	T
mean	3.85 mV	13.43 mV
MNCS_CV (median nerve)	P	T
mean	50.32 m/s	31.51 m/s
MNCS_OL (median nerve)	P	T
mean	4.9 ms	3.4 ms
MNCS_APA (median nerve)	P	T
mean	5 mV	4.54 mV
SNCS_CV (median nerve)	P	T
mean	35.24 m/s	20.08 m/s
SNCS_APA (median nerve)	P	T
mean	7.52 mV	9.82 mV

## Data Availability

The data generated and/or analyzed during the current study are available from the corresponding author upon reasonable request.

## Supplementary Materials

The following supporting information can be downloaded at: https://www.mdpi.com/article/10.3390/bioengineering12090945/s1. Figure S1: Preparation of sural nerve graft; Figure S2: Predegenerated gratf after reconstruction; Figure S3: Microsurgical nerve reconstruction.

## Abbreviations

The following abbreviations are used in this manuscript:

HVI	Hand volume index
HMSI	Hand muscle strength index
NCV	Nerve conduction velocity
ENG	Electroneurography
MNCS_APA	Motor Nerve Conduction Study—Action Potential Amplitude
MNCS_APAA	Motor Nerve Conduction Study—Action Potential Amplitude Assessment
SUM	Medical University of Silesia
kDa	kilo Daltons
NGF	Nerve growth factor
BDNF	Brain-derived neurotropic factor
CNTF	Ciliary neurotrophic factor
IGF-I	Insulin-like growth factor 1
IGF-II	Insulin-like growth factor 2
S-100	Family of multigenic group of nonubiquitous cytoplasmic homologous intracellular Ca^2+^ binding proteins with EF hand motifs and significant structural similarities at both protein and genomic levels
PLP	Proteolipid proteins
