# Supplementary figures and images for: The Influence of a Predegenerated Autological Nerve Graft on the Results of Peripheral Nerve Repair in the Upper Extremities After Injuries
Source: Bioengineering (Basel). 2025 Aug 31;12(9):945. doi: 10.3390/bioengineering12090945 (PMC12467786; doi:10.3390/bioengineering12090945)

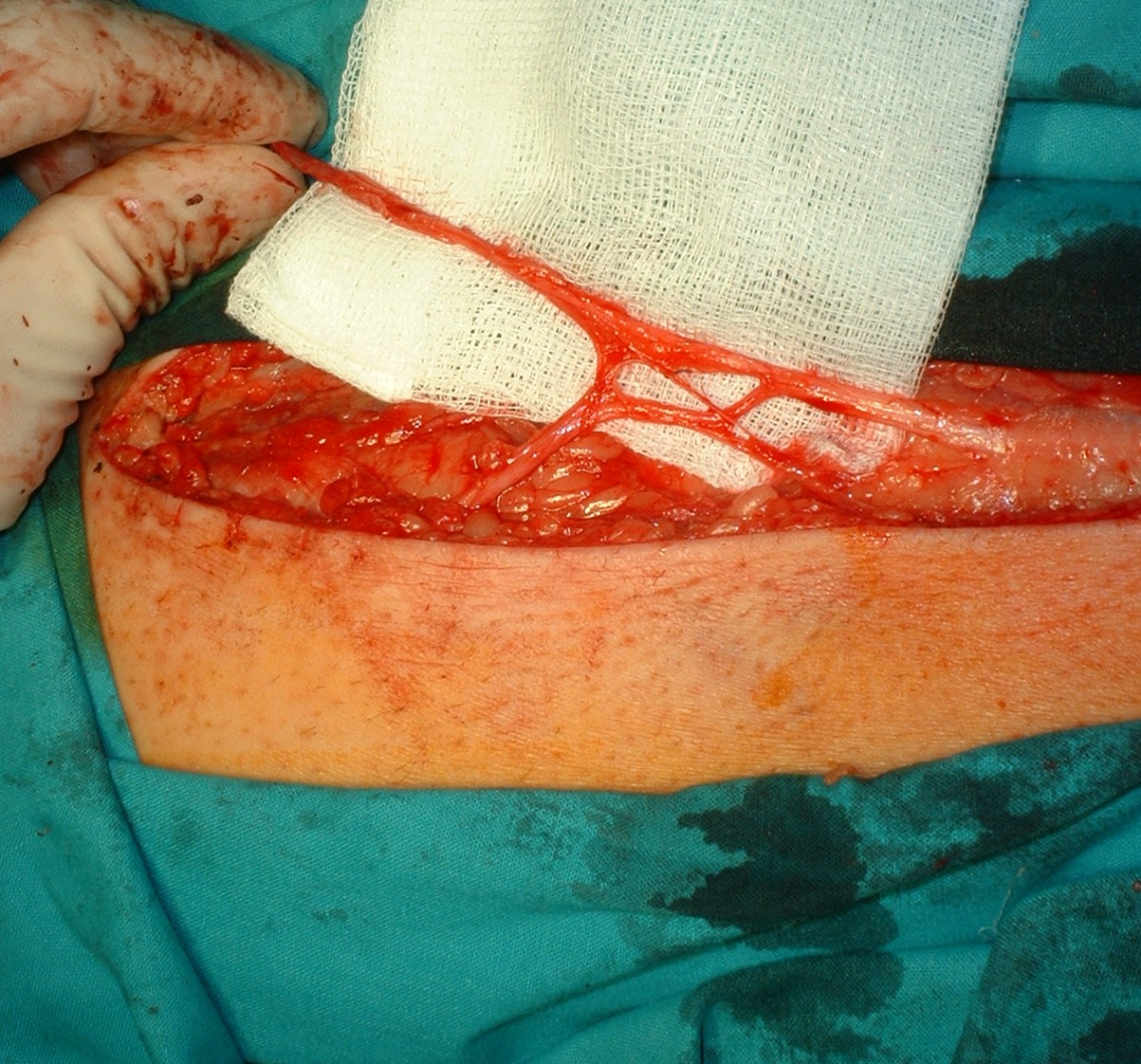

Supplement: Supplementary file 1 [file bioengineering-12-00945-s001.zip › Figure S1 Preparation of sural nerve graft.tif]

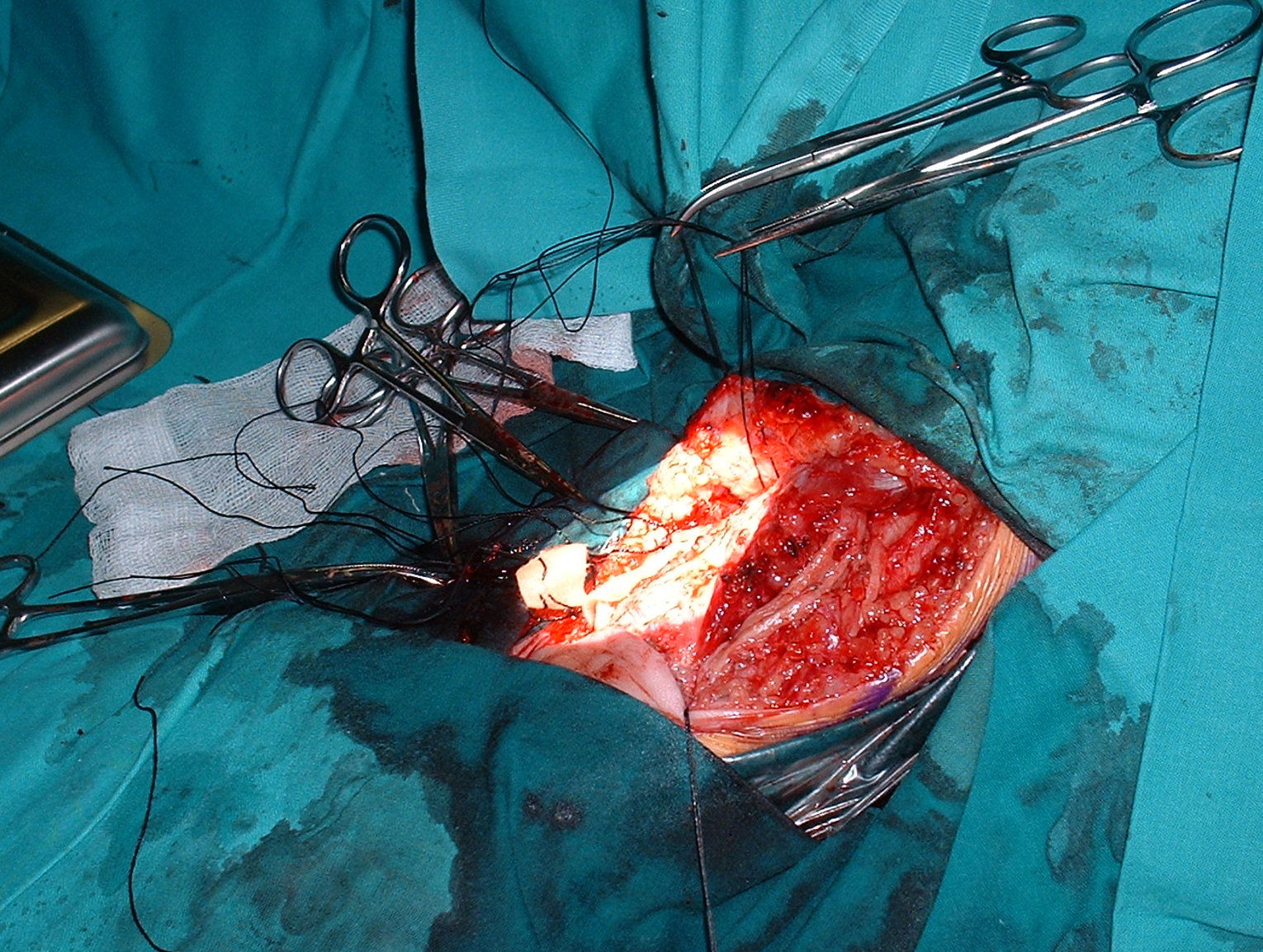

Supplement: Supplementary file 1 [file bioengineering-12-00945-s001.zip › Figure S2 Predegenerated gratf after reconstruction.tif]

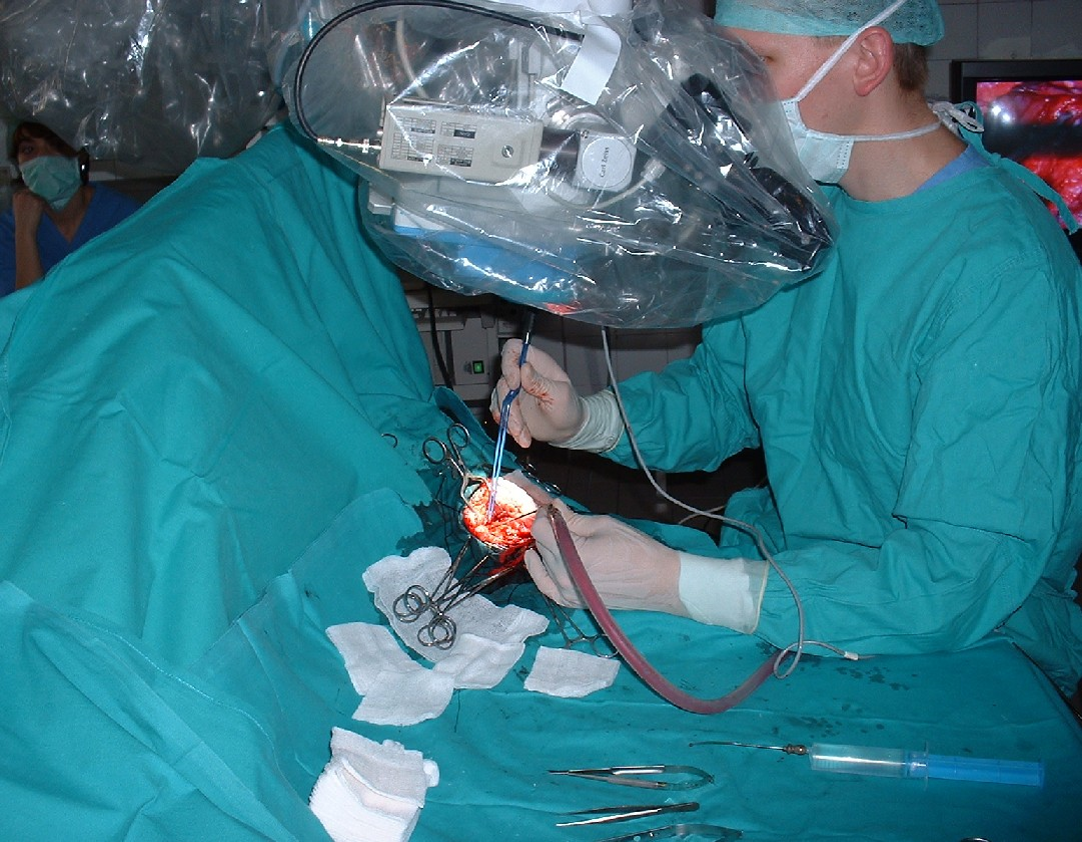

Supplement: Supplementary file 1 [file bioengineering-12-00945-s001.zip › Figure S3 Microsurgical nerve reconstruction.tif]
